# Lipid metabolic pathways converge in motor neuron degenerative diseases

**DOI:** 10.1093/brain/awz382

**Published:** 2019-12-18

**Authors:** Olivia J Rickman, Emma L Baple, Andrew H Crosby

**Affiliations:** Medical Research (Level 4), RILD Wellcome Wolfson Centre, University of Exeter Medical School, Royal Devon and Exeter NHS Foundation Trust, Barrack Road, Exeter, EX2 5DW, UK

**Keywords:** HSP, MND, cholesterol, mitochondria, lipidome imbalance

## Abstract

Motor neuron diseases (MNDs) encompass an extensive and heterogeneous group of upper and/or lower motor neuron degenerative disorders, in which the particular clinical outcomes stem from the specific neuronal component involved in each condition. While mutations in a large number of molecules associated with lipid metabolism are known to be implicated in MNDs, there remains a lack of clarity regarding the key functional pathways involved, and their inter-relationships. This review highlights evidence that defines defects within two specific lipid (cholesterol/oxysterol and phosphatidylethanolamine) biosynthetic cascades as being centrally involved in MND, particularly hereditary spastic paraplegia. We also identify how other MND-associated molecules may impact these cascades, in particular through impaired organellar interfacing, to propose ‘subcellular lipidome imbalance’ as a likely common pathomolecular theme in MND. Further exploration of this mechanism has the potential to identify new therapeutic targets and management strategies for modulation of disease progression in hereditary spastic paraplegias and other MNDs.

## Teaser

While molecular studies commonly show lipid abnormalities in monogenic motor neurone diseases, there is no clarity regarding the key functional pathways involved. Rickman *et al.* review the evidence to propose ‘subcellular lipidome imbalance’ in specific lipid pathways as a common pathological mechanism in these disorders.

## Motor neuron diseases

**Figure 1:**
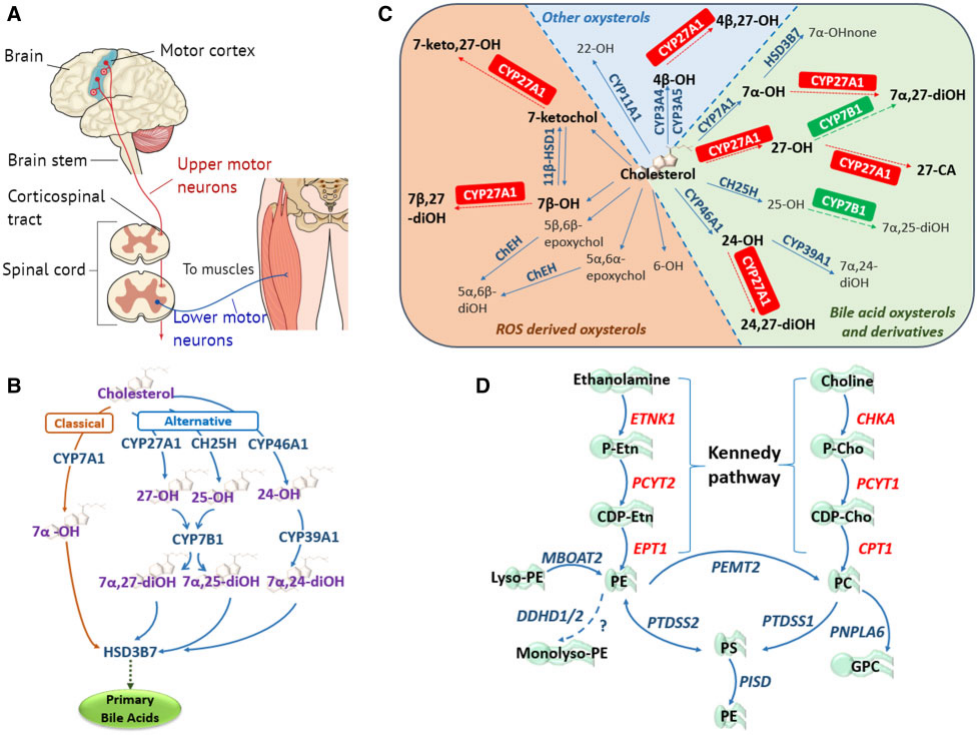
**The primary motor neuron components and lipidome pathways in MND/HSP.** (**A**) Simplified schematic of the core components of the primary motor pathway, including upper and lower motor neurons, for the transmission of voluntary commands from the primary motor cortex to skeletal muscles. (**B**) Classic and alternative pathways through which cholesterol can be converted into primary bile acids. The classical pathway is initiated by cholesterol 7α-hydroxylase (CYP7A1) located in the endoplasmic reticulum (ER) of the liver, catalysing the conversion of cholesterol to 7α-OH. The alternative pathways are initiated by three enzymes: (i) the mitochondrial sterol 27-hydroxylase (CYP27A1) requiring mitochondrial cholesterol import, primarily via the ER and lipid droplets, mediated by steroidogenic acute regulatory protein (StAR) and StAR-related lipid transfer domain (StarD) proteins (Norlin *et al.*, 2000; Scharwey *et al.*, 2013); (ii) the liver microsomal sterol 25-hydroxylase (CH25H); and (iii) the brain sterol 24-hydroxylase (CYP46A1), forming 27-OH, 25-OH and 24-OH, respectively. Oxysterol 7α-hydroxylase (CYP7B1) catalyses the 7α-hydroxylation of both 25- and 27-OH to 7α,25- and 7α,27-diOH, whereas liver microsomal oxysterol 7α-hydroxylase II (CYP39A1) catalyses the 7α-hydroxylation of 24-OH to 7α,24-diOH (Li-Hawkins *et al.*, 2000). In the liver the 7α-hydroxylated oxysterols converge in the common bile acid pathway, initiated by 3β-hydroxysteroid dehydrogenase (HSD3B7), where they are further modified to form the primary bile acids. (**C**) Enzymatically (blue and green) and ROS (orange) derived oxysterols, highlighting the importance and multiple enzymatic roles of the mitochondrial sterol 27-hydroxylase (CYP27A1) in oxysterol metabolism. CA = cholestanoic acid; ChEH = cholesterol epoxide hydrolase; HSD = hydroxysteroid dehydrogenase; none = cholestenone; ROS = reactive oxygen species. Figure adapted from Mutemberezi *et al.* (2016). (**D**) Biosynthetic pathways for phosphatidylethanolamine (PE) synthesis. The primary routes of PE synthesis include the Kennedy pathway (enzymes shown in red) and the phosphatidylserine (PS) decarboxylase pathway in mitochondria. PE is formed from ethanolamine via the CDP-ethanolamine branch of the Kennedy pathway comprising three enzymatic steps. Two additional pathways of PE production: the acylation of lyso-PE catalysed by lyso-PE-acyltransferase (MBOAT2), and the synthesis from PS via PS-synthase-2 (PTDSS2). The CDP-choline branch of the Kennedy pathway forms phosphatidylcholine (PC), also via three enzymatic steps. PE and PC synthesized via the Kennedy pathway is catalysed by PS-synthase-1 and 2 (PTDSS1/2) to PS, which can be transported to the inner mitochondrial membrane where PSD (encoded by *PISD*) decarboxylates it to PE. Phosphatidylethanolamine *N*-methyltransferase 2 (PEMT2) methylates PE to form PC. DDHD1/2 may also play a role in maintaining PE homeostasis.

Motor neuron diseases (MNDs) are a large genetically and clinically heterogeneous group of incurable neurological diseases characterized by the progressive degeneration of upper and/or lower motor neurons (Fig. 1A). The clinical presentation and classification in MND is largely dependent on whether upper motor neurons, lower motor neurons, or both, are primarily involved (Statland *et al.*, 2015) (Fig. 1A). Upper motor neuron malfunction may primarily impair the modulation of muscular movement resulting in muscle stiffness and spasticity, the cardinal clinical feature seen in the hereditary spastic paraplegias (HSPs). HSP was first described by Strumpell and Lorrain in the late 19th century and was considered to be a small group of Mendelian disorders. However, subsequent advancements in our understanding of the genetic architecture of HSP have led to it being recognized as one of the most genetically heterogeneous of inherited disorders. The condition may be divided into ‘pure HSP’ in forms of the disease primarily entailing lower limb spasticity, and ‘complex HSP’ when limb spasticity is accompanied by other neurological (or non-neurological) clinical signs (Noreau *et al.*, 2014). HSP subtypes were originally designated spastic paraplegia (SPG) prefixes to demarcate genetic loci in approximate numerical order of their discovery. However, due to the large number of gene loci, the rate of gene discovery, and the complex clinical presentations associated with many spasticity-associated disorders, genes associated with HSP identified in more recent years have no longer used this classification system.

Lower motor neuron malfunction may directly compromise muscular innervation, leading to muscular atrophy, weakness and hyporeflexia. The distal hereditary motor neuropathies (dHMNs) comprise a heterogeneous group of primarily lower motor neuron diseases that share the common feature of a length-dependent motor neuropathy, and progressive distal muscle weakness. Weakness usually presents in the lower limb extremities in the second or third decade of life, progressing to the distal muscles of the upper limbs (Irobi *et al.*, 2004). There are currently ∼30 genes known to be responsible for dHMNs (Irobi *et al.*, 2004; Rossor *et al.*, 2012), with many forms also displaying sensory abnormalities and/or an upper motor neuron component, and as such often overlap with other forms of MND. This overlap includes Charcot-Marie-Tooth disease (CMT, also known as hereditary motor and sensory neuropathy; HMSN), which comprises an additional distinct group of lower motor neuron conditions with a sensory component. CMT is the most common inherited MND with a prevalence of ∼1 in 2500 with >80 distinct genetic causes identified, many of which may also cause other forms of MND (Szigeti and Lupski, 2009; Magy and Vallat, 2015).

Amyotrophic lateral sclerosis (ALS), occurring with a prevalence of 3–5/100 000 in Europe and the USA (Brown and Al-Chalabi, 2017), is associated with both upper and lower motor neuron involvement. The primary symptom of ALS entails motor dysfunction, although ∼50% of affected individuals also develop other cognitive and behavioural impartments during the course of the disease. In ALS, death typically occurs within 3–5 years of diagnosis, although affected individuals may live up to 40 years after the onset of symptoms. Up to 25% of ALS cases have a family history of the disease, with mutations in *C9orf72* and *SOD1* together accounting for ∼40% of familial ALS cases (Rosen *et al.*, 1993; Ajroud-Driss and Siddique, 2015). While the majority of individuals display no clear monogenic basis, most ‘sporadic’ cases are considered likely to have a more complex genetic involvement (Benatar *et al.*, 2016).

### Genetic studies in motor neuron diseases

By providing crucial insight into the underlying molecular abnormalities causative of a disease, advancements in genomic technologies have enabled numerous important discoveries in many fields of biology and medicine in recent years. In MNDs, genetic studies have identified mutations in hundreds of genes and molecules that may lead to this family of conditions. The extreme genetic heterogeneity in MND is exemplified by the HSPs, for which >80 distinct genetic causes have been defined (Das Bhowmik *et al.*, 2018), with many other genetic disorders also described in which HSP/spasticity is a key diagnostic feature. The genes responsible for these conditions have been associated with a plethora of cellular roles, extensively reviewed elsewhere (Blackstone, 2012; Fink, 2013; Lo Giudice *et al.*, 2014).

A common feature of familial MNDs involves the notably variable penetrance, age of onset, progression and specific clinical nature of disease. Not infrequently, more than one clinical form of MND may be present in the same family, associated with the same genetic variant. Similar clinical variability may also be seen between families with phenotypically distinct presentations of MND, associated with variants within the same gene. This indicates that common cellular deficits and pathomolecular mechanisms are likely to underlie malfunction and degeneration in a range of neuronal cell types. Abnormalities within numerous subcellular cascades and biological processes will undoubtedly drive neurodegenerative outcomes seen in MNDs. However, it is clear from the large numbers of genes involved, and their overlapping phenotypes, that many will map to specific subcellular processes, which may be commonly impaired by abnormalities in a wide array of molecules.

Genetic, molecular, cell and animal studies have identified dysfunctional lipid metabolic outcomes as a common theme in MND-associated molecules. Additionally, a sizeable group of gene mutations have been associated with both functionally and morphologically abnormal subcellular organelles, in particular the endoplasmic reticulum (ER), endo/lysosomal and mitochondrial compartments. However, because of the largely poorly-defined subcellular roles of many of the molecules involved, it has proven challenging to define specific biological processes that may commonly malfunction to give rise to motor neuron degeneration. This review aims to highlight evidence defining molecular imbalances within two related specific lipid cascades, manifesting through subcellular organelle interfaces (in particular the mitochondrial-ER interface), as common pathomolecular themes in HSP and other MNDs.

## Cholesterol and bile acid (oxysterol) biosynthesis

Cholesterol is a type of lipid hydrocarbon with its name derived from ancient Greek [chole (bile), and stereos (solid)], due to it first being identified as a component of gallstones (de Oliveira Andrade, 2016). The specific hydrocarbon properties of cholesterol underpin its role as an important constituent of cellular membranes, where it is typically found in high concentration (Goluszko and Nowicki, 2005). This is particularly the case in the brain, the most cholesterol-rich organ (Zhang and Liu, 2015), in which it is an integral component of myelin accounting for ∼80% brain cholesterol, vital for the electrical insulation of neurons for rapid signal transduction (Saher and Stumpf, 2015). However, as peripheral circulating cholesterol does not cross the blood–brain barrier, the brain must synthesize its own cholesterol. As in adults, the rate of synthesis exceeds requirements, excess cholesterol must be extruded from the brain to prevent toxic accumulation (Dietschy and Turley, 2004). Cholesterol metabolism involves the bile acid synthesis pathway, a cascade of 16 distinct enzymes with primary sites of action in the ER, mitochondria, peroxisomes and cytosol (Hedlund *et al.*, 2001; Russell, 2003), which enable bile acid extrusion via a series of intermediate ‘oxysterol’ derivatives (Fig. 1B). In the liver, cholesterol is converted into primary bile acids via the ‘classical’ pathway, involving CYP7A1 (Norlin *et al.*, 2000). In the brain, neuronal cholesterol clearance is achieved via an ‘alternative’ branch of the bile acid synthesis pathway, which involves three parallel arms; CYP27A1 in mitochondria, and CH25H (and subsequently CYP7B1) and CYP46A1 in the ER, the latter converting cholesterol into 24-hydroxycholesterol (24-OH), which provides the major pathway for cholesterol elimination from the brain (Lütjohann *et al.*, 1996; Russell, 2009). The various initial arms of the oxysterol cascade then converge with 3β-hydroxysteroid dehydrogenase (HSD3B7), which catalyses the first step of the common bile acid pathway initiating the subsequent steps leading to the primary bile acids chenodeoxycholic and cholic acid (Chiang, 2013).

### Oxysterol imbalance in motor neuron diseases

As well as comprising bile acid intermediates for cholesterol extrusion, the oxysterols entail a large family of lipids (the ‘oxysterome’), which have themselves been found over recent years to be important for a plethora of physiological processes including cell/nuclear receptor-mediated signalling, glucose homeostasis, and immune response (Mutemberezi *et al.*, 2016). In particular, oxysterols are important for regulating levels of cholesterol, which must be constrained within narrow limits within the cell to sustain appropriate membrane permeability, fluidity, and function (Olkkonen *et al.*, 2012). One mechanism of oxysterol regulation involves the processing of sterol regulatory element-binding proteins (SREBPs), transcription factor regulators of cholesterol synthesis and uptake (Radhakrishnan *et al.*, 2007). Oxysterols also bind to and activate liver X receptors (LXRs) to induce cholesterol efflux, and reduce lipoprotein cholesterol uptake (Janowski *et al.*, 1996; Olkkonen *et al.*, 2012). While the precise mechanism of oxysterol transport remains to be determined, a group of conserved proteins, including oxysterol binding protein (OSBP) and OSBP-related proteins (ORPs), bind and traffic oxysterols to elicit multiple cellular processes (Raychaudhuri and Prinz, 2010; Olkkonen *et al.*, 2012). In particular, 27-OH and 24-OH are potent inhibitors of cholesterol synthesis in the brain, and notably alterations in these oxysterols have been implicated in a range of neurodegenerative diseases (Ali *et al.*, 2013; Testa *et al.*, 2016).

A key finding highlighting a central role of altered oxysterol metabolism in motor neuron degeneration is the association of mutations within core enzymatic components of the cascade with MNDs. Mutations in *CYP7B1* were initially defined in patients with an early onset, autosomal recessive form of pure HSP (SPG5) (Tsaousidou *et al.*, 2008); however, more clinically complex forms of HSP (including spastic ataxia and white matter lesions, demyelinating polyneuropathy, optic atrophy, and familial macular dystrophy) have subsequently been infrequently described (Tsaousidou *et al.*, 2008; Arnoldi *et al.*, 2012; Schöls *et al.*, 2017; Marelli *et al.*, 2018). The analysis of CSF and blood plasma in HSP patients with *CYP7B1* gene mutation defined alterations in levels of several oxysterols including the accumulation of metabolic precursor substrates (25-OH and 27-OH), indicative of altered flux through the classical versus alternate arms (Abdel-Khalik *et al.*, 2018; Marelli *et al.*, 2018; Kakiyama *et al.*, 2019).

Mutations in a second enzyme in the oxysterol cascade, CYP27A1, responsible for the immediate precursor enzymatic step to CYP7B1 in the alternate arm of oxysterol pathway, have also been associated with overlapping neurodegenerative outcomes. Mutations in the *CYP27A1* gene result in cerebrotendinous xanthomotosis (CTX), a well-defined rare autosomal recessive lipid storage disorder characterized by tendinous xanthomas, presenile cataract and diarrhoea (Saxena and Pradhan, 2016). While CTX is also characterized by a wide range of neurological presentations including intellectual disability, dementia, ataxia, epilepsy and psychiatric symptoms (Nie *et al.*, 2014), spasticity (and HSP) is considered the cardinal and often the presenting clinical feature, being noted in all CTX patients (Mignarri *et al.*, 2011; Nie *et al.*, 2014). However, unlike the much more restricted catalytic activity of CYP7B1, CYP27A1 is crucial for a wide range of metabolic steps at other positions within the oxysterol cascade (Russell, 2003; Mutemberezi *et al.*, 2016) (Fig. 1C). Therefore, as well as influencing metabolic flux through the alternative (mitochondrial) arm of the oxysterol cascade, the absence of CYP27A1 functionality results in a more widespread impairment of bile acid synthesis. Consistent with this, *CYP27A1* gene mutations responsible for CTX have been shown to result in a global impairment of bile acid metabolism, ultimately leading to the accumulation of tissue cholesterol and cholestenol, characteristic of this disorder (Chen *et al.*, 2011). This more impacting effect on bile acid biosynthesis of CYP27A1 malfunction is likely to account for the broader array of clinical features seen in CTX, compared with the more specific oxysterol consequences in CYP7B1-associated HSP.

To investigate the oxysterol metabolic pathways in more detail, and learn more about their molecular roles and the phenotypical consequences of gene mutation, a wide range of knockout models eliminating the function of enzymes within the bile acid cascade have been generated. These models provided important insights into oxysterol metabolism and function, and identify altered flux and complex compensatory metabolic feedback mechanisms within the various oxysterol metabolic cascades. While mutations of *CYP46A1* have yet to be associated with monogenic disease in humans, *Cyp46a1* knockout mice display altered cholesterol metabolism resulting in neurodegeneration and motor deficits, which may be reversed upon restoration of CYP46A1 activity (Lund *et al.*, 2003; Boussicault *et al.*, 2016). Thus, it is clear that disruption of the metabolic processes of the alternative arms of the bile acid metabolic cascade may form a common biomolecular theme in neurodegenerative disease, in particular MND.

## Phosphatidylethanolamine biosynthesis

Glycerophospholipids including phosphatidylethanolamine (PE) and phosphatidylcholine (PC) are the primary lipid species of eukaryotic cell membranes (Gibellini and Smith, 2010; Vance and Tasseva, 2013). PE is crucial for providing structural support to cellular membranes and sustaining the function of intrinsic membrane proteins, and is involved in a wide range of cellular processes including in anti-inflammatory, proapoptotic, autophagic, and cell surface signalling (Menon *et al.*, 1993; Momchilova and Markovska, 1999; Ichimura *et al.*, 2000; Okamoto *et al.*, 2004; Braverman and Moser, 2012; Rockenfeller *et al.*, 2015). PE is also integral for maintaining membrane architecture in the mitochondrion, and during cell division and membrane fusion processes, via its unique biophysical properties (Signorell *et al.*, 2009). As with cholesterol, PE is particularly enriched in the brain where it accounts for ∼45% of total phospholipid content, compared to ∼25% in other cell types (Vance and Tasseva, 2013). PE biosynthesis involves several different pathways, in particular the CDP-ethanolamine pathway (often referred to as the Kennedy pathway) and through the decarboxylation of phosphatidylserine (PS) via the mitochondrial phosphatidylserine decarboxylase (PSD, encoded by *PISD*), accounting for the majority of mitochondrial PE (Vance and Tasseva, 2013). Kennedy pathway PE biosynthesis involves three enzymatic steps catalysed by *ETNK1*, *PCYT2*, and *EPT1*, undertaken in the ER (Fig. 1D). A parallel arm of the cascade generates PC from choline via the sequential activity of three enzymes encoded by three genes; *CHKA*, *PCYT1* and *CPT1* (Fig. 1D) (Gibellini and Smith, 2010). Other biosynthetic pathways are also known to involve PE pathway molecules, involving *PTDSS2*, *MBOAT2*, *PEMT2* and *PNPLA6* (Vance, 2013; Vance and Tasseva, 2013; Hermansson *et al.*, 2016), a number of which involve the transfer of substrates between ER and mitochondrial compartments (Aaltonen *et al.*, 2016; Tatsuta and Langer, 2017); as such the interface between ER and mitochondria serves as a crucial hub for phospholipid biosynthesis.

### Dysfunctional phosphatidylethanolamine biosynthesis in motor neuron diseases

The relevance of aberrant PE biosynthesis to MND is provided by the recent discovery of mutations in core enzymatic components of the biosynthetic cascade associated with these conditions. Mutations in the enzymes catalysing the two precursor steps of PE biosynthesis of the Kennedy pathway (EPT1 and PCYT2) have recently been shown to cause clinically overlapping autosomal recessive forms of complex HSP, involving progressive spastic paraplegia, retinopathy, delayed gross motor development, and gradual decline in motor function, with dysarthria becoming more prominent with disease progression (Ahmed *et al.*, 2017). Additionally, mutations in other arms of the PE/PC metabolic cascade have been associated with HSP. The intracellular phospholipase A_1_ enzymes DDHD1 and DDHD2 hydrolyse phospholipids and are important for lipid transport and metabolism, and intracellular membrane trafficking (Tani *et al.*, 2012). DDHD1 is a cytosolic enzyme, while DDHD2 is thought to be primarily localized to the cytosol, Golgi apparatus and the ER (Maruyama *et al.*, 2018). The yeast homologue of DDHD1/2, Ddl1, has been shown to hydrolyse PE to monolysophosphatidylethanolamine, and play an important role in mitochondrial phospholipid remodelling (Yadav and Rajasekharan, 2016). Mutations in *DDHD1* are associated with a range of motor neuron phenotypes including both pure and complex forms of autosomal recessive HSP (SPG28) associated with retinopathy, among other clinical signs (Bouslam *et al.*, 2005; Miura *et al.*, 2016; Dard *et al.*, 2017), as well as juvenile ALS (Wu and Fan, 2016). Notably, tissue from patients with *DDHD1*-associated MND exhibit mitochondrial abnormalities and reduced energy metabolism (Mignarri *et al.*, 2016). Similarly, mutations in *DDHD2* have been associated with an autosomal recessive early-onset spastic paraplegia accompanied by intellectual disability (SPG54) (Schuurs-Hoeijmakers *et al.*, 2012; Gonzalez *et al.*, 2013). Consistent with a role in PE metabolism, previous studies identified lipid droplet accumulation in the brains of SPG54 patients, and in *DDHD2* knockout mice (Fei *et al.*, 2011; Inloes *et al.*, 2018). Studies of *DDHD2* knockout mice also revealed concomitant abnormalities in other arms of the PE metabolic cascade including reduced CL levels, and identified a role for DDHD2 in preventing reactive oxygen species (ROS) production and protecting cells against oxidative stress, although the precise mechanistic basis of these functions remains obscure (Maruyama *et al.*, 2018). Additionally, mutations in another core metabolic component of the PE/PC cascade, *PNPLA6* (also known as *NTE*; neuropathy target esterase), which catalyses the conversion of PC to lysophosphatidylcholine (LPC) and LPC to glycerophosphocholine (GPC), have been associated with a range of HSP presentations, as well as other more complex neurological and retinal disorders (Synofzik *et al.*, 2013; Topaloglu *et al.*, 2014; Hermansson *et al.*, 2016). Together, the association of mutations in a range of enzymes involved in PE/PC metabolism with HSP/MNDs therefore highlights the importance of the integrity of this pathway for normal motor neuron maintenance and function. Furthermore, it is also notable that MND outcomes associated with disruptions within the PE/PC metabolic cascade are commonly accompanied by retinal/retinopathy phenotypes, identifying a particular vulnerability of both retinal as well as neuronal cell types to disruptions in this metabolic cascade.

## Mitochondrial-associated endoplasmic reticulum membrane

**Figure 2:**
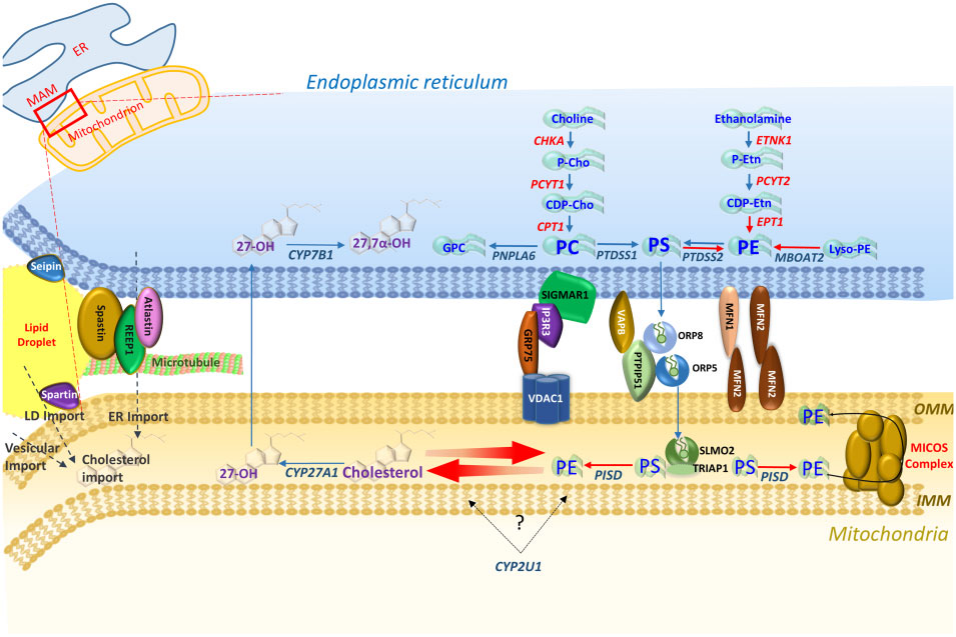
**PE and oxysterol pathways and the mitochondria-associated ER membrane (MAM).** Overviewing enzymes in each pathway, three of the four main tethering complexes of the MAM-mitochondrial region, and other HSP/MND-associated molecules with putative roles in these areas. PS synthesized in the ER membrane is then transported across the MAM via ORP5 and ORP8 that interact with protein tyrosine phosphatase interacting protein 51 (PTPIP51) on the outer mitochondrial membrane (OMM) (Galmes *et al.*, 2016). PS is then transported to the inner mitochondrial membrane (IMM) via the SLMO2-TRIAP1 lipid transfer complex for delivery to PSD where it is decarboxylated to PE. The mitochondrial contact site and cristae organizing system (MICOS) complex spans the mitochondrial membrane and is crucial for the distribution of PE to the OMM and ER (Tatsuta and Langer, 2017).

Many previous studies document physical membrane-membrane interactions between subcellular organelles including the ER, mitochondria, Golgi intermediate compartments, peroxisomes, and plasma membranes, to enable a myriad of biomolecular processes (Voeltz *et al.*, 2002; Hoepfner *et al.*, 2005; Voelker, 2005; Dimmer and Rapaport, 2017). The unique subdomain of the ER that associates with mitochondria forms a region termed as the ‘mitochondria-associated ER membrane’ (MAM) domain (Vance, 2014), a dynamic structure readily influenced by the subcellular microenvironment including calcium, and notably cholesterol, levels (Hajnóczky *et al.*, 2006; Fujimoto *et al.*, 2012). Over recent years it has become evident that ER-mitochondrial contact sites are crucial for a range of fundamental cellular functions, including the maintenance of mitochondrial dynamics and DNA integrity, the regulation of cellular calcium homeostasis, apoptosis, and autophagy. As may be expected from the importance of these overlapping cellular processes, defects in tethering the ER to mitochondria are likely to contribute to the pathogenesis of a range of human diseases (Vance, 2014). Recent studies have identified four main molecular tethering mechanisms mediating mitochondrial-ER connections (Fig. 2): (i) mitochondrial mitofusin 1 (MFN1) interacting with ER mitofusin 2 (MFN2), with tethering also via MNF2-MFN2 homodimers mediated through a pool of the molecule present on the mitochondrial membrane (Filadi *et al.*, 2018); (ii) mitochondrial tyrosine phosphatase-interacting protein 51 (PTPIP51) interacting with ER vesicle associated membrane protein-associated protein B (VAPB) (Gomez-Suaga *et al.*, 2017); (iii) mitochondrial voltage-dependent anion channel 1 (VDAC1) complex formation important for calcium ion exchange with 75 kDa glucose-regulated protein (GRP75) and Sigma 1 receptor (SIGMAR1), and ER 1,4,5-triphosphate receptor 3 (IP3R3); and (iv) ER B-cell receptor-associated protein 31 (BAP31) complex formation with mitochondrial fission protein FIS1 (fission 1 homologue) and the phosphofurin acidic cluster sorting protein-2 (PACS-2), important for apoptotic processes (Bernard-Marissal *et al.*, 2018).

MAM-mitochondria contact sites are also crucial for the synthesis and shuttling of lipids between the ER (their primary site of synthesis) and mitochondria to maintain synthesis pathways, an interplay that requires a host of protein tethers, transporters, chaperones, and scaffolding molecules (Vance, 2014). Additionally the MAM-mitochondrial region is also enriched in cholesterol, PE and lipases crucial for biosynthesis, and transport machinery for the import of PS to mitochondria for decarboxylation to PE (Vance, 2014; Tatsuta and Langer, 2017). MAM-mitochondria contact sites therefore provide a hub for phospholipid biosynthesis and exchange between subcellular compartments, and serve as a conduit through which lipidome pathways may converge. In this regard, and given the overlapping MND phenotypes associated with their metabolic pathways, it is notable that there is growing evidence for interplay and correlated feedback between the PE and cholesterol metabolic cascades. Previous studies show that cholesterol levels negatively regulate the association between MAMs and mitochondria, which in turn directly influence PE biosynthesis (Fujimoto *et al.*, 2012). Thus, both bile acid and PE lipid biosynthetic pathways implicated in MND phenotypes appear to interface at the MAM-mitochondrial contact region.

## ‘Subcellular lipidome imbalance’ as a common linking theme in HSP and motor neuron disease

The above studies underscore the critical importance of the proper maintenance of both oxysterol and PE/PC molecular pathways for motor neuron survival, implicating subcellular lipidome imbalances within these (and likely other inter-relating lipid) pathways as a common driver of motor neuron (and retinal) phenotypes. Importantly, however, the enzymes within these cascades do not function in isolation; catalysis involves a complex and intricate series of auxiliary molecules and subcellular processes (including chaperones, co-factors, receptors, transporters or structural matrices) to execute the enzymatic steps themselves, and for substrate shuttling between subcellular compartments. Similar to mutations within the core enzymatic components themselves, a likely outcome of gene mutation leading to malfunction of any of the auxiliary components is impaired metabolic efficiency, resulting in lipidome pathway imbalances. However, defining the relevance to these pathways of the many other molecules known to be mutated in HSP/MND has been problematic. This may be explained in a number of ways. In particular, biochemical imbalances associated with mutations in pathway auxiliary molecules may typically be more modest in nature, due to the integrity of the core enzymatic components (i.e. the enzymes themselves) being essentially preserved. As such biochemical analyses of plasma or CSF metabolites, which may be relevant for disorders involving the core metabolic enzymes, may not readily identify biomarker abnormalities associated with gene mutations affecting pathway auxiliary molecules. Difficulties in defining a clear impact of pathway auxiliary molecules on these cascades may be further compounded by their outcomes being primarily subcellular, and the limited availability of methodologies to probe resulting biomarker imbalances, particularly when the consequences of gene mutations may be specific to the brain. Additional difficulties in identifying common biomolecular themes in MNDs stem from the complex range of neurological (and non-neurological) clinical presentations, and disparate clinical diagnoses, associated with abnormalities in each cascade. This may relate to additional functional activities of the particular molecules involved, as is the case for CYP27A1 mutation in CTX, masking the association of specific phenotypical presentations (i.e. HSP) with disruption of a particular molecular cascade.

However, subtle but catastrophic subcellular oxysterol/lipidome imbalance may arise in these metabolic cascades due to gene mutations in pathway auxiliary molecules, and a growing body of evidence exists to indicate that many molecules known to cause HSP/MND phenotypes are likely to have related and important functional roles in this area. One example entails mutations in ER lipid raft-associated protein 2 (ERLIN2), which result in autosomal recessive pure as well as complex forms of HSP associated with cognitive decline (SPG18) (Alazami *et al.*, 2011; Tian *et al.*, 2016), and autosomal dominant pure HSP. The Erlin molecules (ERLIN1 and 2) are ∼40-kD ER proteins that were originally characterized by their fractionation in cholesterol-enriched, detergent-resistant membrane derivatives (Browman *et al.*, 2006). A series of cell localization and expression studies define an important role for ERLIN2 in the regulation of SREBP transcription factors, central to the role in regulating cholesterol and fatty acid homeostasis (Huber *et al.*, 2013). Oxysterols have also been shown to regulate SREBPs, in addition to LXRs, in order to govern cholesterol levels via a tightly controlled feedback mechanism (Olkkonen *et al.*, 2012).

As well as being responsible for an autosomal recessive form of lipodystrophy, mutations in the Berardinelli-Seip congenital lipodystrophy 2 (*BSCL2*) gene encoding seipin may also result in a number of autosomal dominant forms of MND including HSP with amyotrophy (Silver syndrome; SPG17), CMT2, and dHMN type V (Windpassinger *et al.*, 2004; Cen *et al.*, 2015). A range of molecular studies have defined a clear role for seipin as an ER transmembrane protein located in the vicinity of lipid droplet budding sites, involved in lipid droplet formation (Szymanski *et al.*, 2007; Wang *et al.*, 2016) and phospholipid metabolism (Ding *et al.*, 2018), indicating a clear role for seipin in lipid metabolic processes. Lipid droplets are cellular stores primarily of neutral lipids, such as triglycerides and cholesterol esters, for use in times of need (Welte, 2015). The droplets are formed from the ER where they are packaged in a phospoholipid bilayer, and notably altered PE levels appear to play a role in the regulation of lipid droplet emergence from the ER (Liu *et al.*, 2015; Welte, 2015; Choudhary *et al.*, 2018).

Mutations in another HSP molecule, spartin (SPG20), are associated with an autosomal recessive complex form of HSP originally identified in the Amish community and subsequently described elsewhere, encompassing neurological as well as non-neurological features (Patel, 2002; Ciccarelli *et al.*, 2003; Bizzari *et al.*, 2017; Dardour *et al.*, 2017). Given this complex clinical presentation, it is perhaps not surprising that spartin has been linked to several subcellular processes (Bakowska *et al.*, 2005, 2007). However studies of *Spg20* knockout mice define clear outcomes of loss of spartin function on lipid metabolic processes, identifying increased lipid droplet numbers as a key finding (Eastman *et al.*, 2009). Other studies further support a role for spartin in lipid dynamics showing that as well as co-localizing with the endosomal cell compartment, endogenous spartin clusters around lipid droplets, a proportion of which may reside in the vicinity of mitochondria (Edwards *et al.*, 2009). Additional studies by other workers also define interaction between spartin and TIP47 (perilipin 3), a protein crucial to lipid droplet formation (Eastman *et al.*, 2009; Nose *et al.*, 2013).

A range of potential functions, in particular microtubule severing (Salinas *et al.*, 2007), have been identified for spastin encoded by the *SPAST* gene, including a clear link with lipid metabolism (Papadopoulos *et al.*, 2015). *SPAST* gene mutation is the most common cause of autosomal dominant pure HSP (SPG4), although in-depth studies of larger patient cohorts have also associated gene mutation with more complex forms of HSP with bulbar dysfunction, and sensory and lower motor neuron signs (McDermott *et al.*, 2006; Sartucci *et al.*, 2007; Kumar *et al.*, 2012). Previous studies identify two spastin isoforms generated from alternative transcription initiation codons (M1 and M87), which appear to possess distinct functional roles with the long spastin isoform (spastin-M1) being predominantly expressed in neurons (Claudiani *et al.*, 2005; Connell *et al.*, 2009; Papadopoulos *et al.*, 2015). The unique N-terminal region of spastin-M1 contains a hydrophobic motif required for ER membrane association, and for molecular interaction with binding partner molecules receptor expression enhancing protein 1 (REEP1) and atlastin-1, which mediate ER fusion (atlastin-1) and morphology (REEP1) (Hu *et al.*, 2009; Orso *et al.*, 2009; Park *et al.*, 2010); both of these molecules are also mutated to cause forms of HSP. Studies of the spastin-M1 isoform show that it is transported from the ER to pre- and mature lipid droplets mediated by the N-terminal hydrophobic motif, and that expression levels of this isoform also influence the number and size of lipid droplets. Similar roles in lipid droplet regulation were also identified in studies of orthologous spastin in *Drosophila* (Dspastin, *spas*) and worm (*Caenorhabditis elegans*; *spas-1)*, defining an evolutionarily conserved role for spastin as a positive regulator of lipid metabolism (Papadopoulos *et al.*, 2015). Consistent with this, roles in lipid droplet size regulation have also been defined for spastin binding partner atlastin-1 (*ATL1*) (Klemm *et al.*, 2013), mostly responsible for autosomal dominant pure HSP (SPG3A), although more complex HSP clinical presentations have also been described associated with *ATL1* gene mutation (Zhao and Liu, 2017). A third molecular partner in the functional complex involving spastin and atlastin is REEP1, in which mutations have been associated with autosomal dominant HSP (SPG31), autosomal dominant dHMN type VB (HMN5B), and autosomal recessive form of congenital axonal neuropathy and diaphragmatic palsy (Lim *et al.*, 2015). *Reep1* null mice also display lipid droplet abnormalities, in addition to prominent motor neuron signs (Renvoisé *et al.*, 2016). Importantly, the REEP1 protein sequence encompasses subdomains for both mitochondrial and ER localization and REEP1 has been shown to be located within MAM-mitochondria contact sites where it facilitates organellar interaction. Crucially, this function is abrogated in the presence of REEP1 disease-associated mutations (Lim *et al.*, 2015). Together these findings define a potential functional role for the spastin-atlastin-REEP1 complex in lipid transactions at the ER-mitochondrial interface.

Consistent with the notion that molecular abnormalities at the mitochondrial-ER interface may impair fundamental functions including lipid metabolism, so leading to MND, is the finding of CMT, dHMN and HSP-related phenotypes correlated with MAM-mitochondria tethering molecules. Mutation in *MFN2*, a key MAM-mitochondria linking protein, has been associated with a broad range of motor neuron phenotypes including CMT (CMT2A2), HMSN with pyramidal features (HMSN V), HMSN with optic atrophy (HMSN VIA) and/or cognitive impairment, and spasticity (Chung *et al.*, 2006; Feely *et al.*, 2011; Ando *et al.*, 2017). In support of this, studies of an *MFN* transgenic mouse model harbouring a pathogenic CMT2A mutation (*MFN2^R94Q^*) identified a notable reduction in ER-mitochondria contacts, a finding that was also replicated in cells of CMT2A patient fibroblasts (Bernard-Marissal *et al.*, 2019). While lipid biosynthesis does not appear to have been specifically investigated, studies of the *MFN2^R94Q^* mouse identified calcium handling defects indicative of MAM-mitochondria malfunction, associated with alterations in the geometry and axonal transport of mitochondria, and ER stress response. Remarkably, treatment of motor neurons with Pre-084, a selective agonist of Sigma 1 receptor (Sig1R, encoded by *SIGMAR1*) also important for ER-mitochondria tethering and function, almost completely prevented neurite degeneration induced by *MFN2^R94Q^*. *SIGMAR1* is also highly expressed in spinal motor neurons, and important for MAM-mitochondria tethering and function (Mavlyutov *et al.*, 2010). *SIGMAR1* gene mutations have again been associated with a range of MND subtypes including early and late-onset ALS and distal HMN with no additional neurological abnormalities (Al-Saif *et al.*, 2011; Ullah *et al.*, 2015; Vettori *et al.*, 2016). As with *MFN2^R94Q^*, studies of cells expressing pathogenic SIGMAR1 mutations (E138Q and E150K) revealed a reduction in total MAM-mitochondria contacts, and incorrect targeting of mutant protein (Vettori *et al.*, 2016). Mutations in another tethering molecule also implicated in lipid metabolism at ER-endosome contact sites result in similar MAM ER-mitochondrial abnormalities, and may give rise to MND phenotypes; vesicle-associated membrane protein B (VAPB) associated with late-onset spinal muscular atrophy and ALS (Nishimura *et al.*, 2004; Dong *et al.*, 2016). While studies have mainly focused on calcium dynamics in these genetic models of disease, the reduced ER-mitochondrial connectivity stemming from all these gene mutations may clearly impact on any functionality mediated via MAM-mitochondria contact, including lipid metabolism.

Aberrant lipid metabolism is also a recurrent theme in many other neurodegenerative diseases in which upper motor neuron involvement and spasticity is an important clinical feature. This includes a family of molecules resulting in neurodegeneration with brain iron accumulation (NBIA), a heterogeneous group of brain iron deposition syndromes in which brain MRI evidence of iron accumulation is accompanied by a variable presentation including dystonia, parkinsonism and neuropsychiatric disturbances. At least 10 genetic causes of NBIA are currently recognized (*PANK2*, *PLA2G6*, *FA2H*, *ATP13A2*, *C19orf12*, *FTL*, *CP*, *C2orf37*, *WDR45* and *CoASY*), a number of which have also been associated specifically with pure or complex HSP forms of HSP (*FA2H*, *PLA2G6*, *ATP13A2* and *C19orf12*) (Dick *et al.*, 2010; Landouré *et al.*, 2013; Allison Gregory, 2017; Estrada-Cuzcano *et al.*, 2017; Tello *et al.*, 2018). Emerging functional data also determine that a number of NBIA genes encode mitochondrial-resident molecules important for lipid metabolism (*PANK2*, *COASY*, *PLA2G6* and *C19orf12*) (Aoun and Tiranti, 2015), and specifically in phospholipid (PE and PC) metabolism (*PLA2G6*) (Deng *et al.*, 2016). Another HSP molecule that may impact directly on lipid pathways is P4502U1 (*CYP2U1*), mutated in both pure and complicated forms of HSP (SPG56) (Kariminejad *et al.*, 2016; Minase *et al.*, 2017; Durand *et al.*, 2018). This molecule comprises a structurally unusual member of the CYP2 gene family, which is broadly known to be important for oxidative metabolism of a range of compounds including steroid hormones and fatty acids (Dhers *et al.*, 2017). While the specific substrate of CYP2U1 remains somewhat unclear, the protein is predominantly expressed in the brain and enriched within microsomal and mitochondrial fractions (Dutheil *et al.*, 2009). While speculative, given these findings, it is tempting to consider that CYP2U1 may also impact upon the mitochondrial lipid processes described in this review.

Thus, taken together, an ever-increasing body of evidence implicates neuronal subcellular lipidome imbalance as a common root cause of HSP and MND outcomes, and potentially other neurodegenerative diseases. In considering these data, this review has focused on studies of monogenic forms of MND, for which clear genetic evidence links gene mutation with disease. However, given the immense number of molecules required for maintenance of the neuronal lipidome, it is conceivable that disruption of subcellular lipid pathways may also underlie more common ‘sporadic’ forms of the condition. Here, however, disease may arise due to a combination of genetic variants in an individual in distinct genes important for lipid metabolic processes. Each particular gene variant may impact lipid metabolism to a modest degree (and thus not cause monogenic disease); however, in combination with other gene variants may have a more profound overall effect on the neuronal subcellular lipidome, resulting in apparently ‘sporadic’ MND.

In the CNS cholesterol, PE and other lipids including sphingolipids have a number of essential functions, particularly in myelination, forming a lipid-rich insulating and biologically-active axonal sheath facilitating the conductance of electrical impulses. The formation and maintenance of myelin involves highly complex cell–cell interactions, reciprocal intercellular signalling events, and precise lipid processing for maintenance of axonal structure and function (Podbielska *et al.*, 2013; Simons and Nave, 2016). Due to their extreme size and the length of their axonal processes, the motor neurons centrally involved in HSP/MND are particularly dependent on normal myelin maintenance and function. As well as the cholesterol/oxysterol and PE/PC pathways defined here, accumulating evidence identifies disturbances in sphingolipid (in particular glycosphingolipid) metabolism in a growing number of neurological diseases including HSP/MND (e.g. FA2H, B4GALNT1) (Harlalka *et al.*, 2013) and GBA2 (Sultana *et al.*, 2015), which may potentially involve disruption of myelin integrity. However, whether a direct link exists between subcellular lipidome imbalance and disordered myelination, leading to neurodegeneration in these and other forms of HSPs/MNDs, remains unclear. It also remains unclear how lipidome imbalance may relate to other well described neurodegenerative pathomolecular processes (e.g. disrupted calcium homeostasis, ROS, and oxidative stress) (Kaufman and Malhotra, 2014; Kim *et al.*, 2015). However, it is notable that abnormal subcellular architecture including altered endosomal, lysosomal and ER morphology (e.g. lysosomal-HSP, ER stress), has been identified in cell studies of a number of the genetically-distinct forms of HSP reviewed above (including spastin, SPG11, SPG15 and REEP1) (Chang *et al.*, 2014; Allison *et al.*, 2017; Branchu *et al.*, 2017). It is tempting to speculate that these subcellular abnormalities, visualized in cell microscopy studies, are the manifestation of subcellular lipidome imbalances potentially within the specific biochemical cascades identified here, which in fact comprise the root and common biochemical cause of disease.

**Table 1:** Genes encoding lipid metabolic and auxiliary pathway components described in this review

Gene	Protein	Function	Phenotype
**Oxysterol metabolism**			
*CYP7B1* ^a^	Oxysterol 7α-hydroxylase	Oxysterol metabolism; 25/27-OH 7α hydroxylation	Complicated HSP – SPG5
*CYP27A1* ^a^	Sterol 27-hydroxylase	Cholesterol 7α hydroxylation	CTX
**Phosphatidylethanolamine metabolism**			
*EPT1* ^a^	Ethanolaminephosphotransferase	Phospholipid metabolism; CDP-ethanolamine to PE	Complicated HSP
*PCYT2* ^a^	Phosphoethanolamine-cytidylyltransferase	Phospholipid metabolism; P-ethanolamine to CDP-ethanolamine	Complicated HSP
*DDHD1* ^a^	Intracellular phospholipase A_1_ DDHD1	Phospholipid metabolism; PE to monolyso-PE; trafficking	Pure/complicated HSP – SPG28; ALS
*DDHD2* ^a^	Intracellular phospholipase A_1_ DDHD2	Phospholipid metabolism; PE to monolyso-PE trafficking	Complicated HSP – SPG54
*PNPLA6* ^a^	Neuropathy target esterase	Phospholipid metabolism; PC to GPC	Complicated HSP – SPG39
**Parallel metabolic cascades**			
*B4GALNT1* ^a^	GM2 synthase	Glycosphingolipid synthesis	Complicated HSP – SPG26
*CYP2U1* ^a^	Cytochrome P450 2UI	Lipid metabolism	Pure/complicated HSP – SPG56
*FA2H* ^a^	Fatty acid 2-hydrolase	Lipid metabolism	Complicated HSP – SPG35; NBIA
*GBA2* ^a^	Non-lysosomal glucosylceramidase	Glycosylceramide metabolism	Complicated HSP – SPG46
*PLA2G6* ^a^	Phospholipase A2	Phospholipid metabolism	Complicated HSP; NBIA
**Auxiliary molecules and/or lipid dysfunction**			
*ATP13A2* ^a^	Cation-transporting ATPase13A2	Membrane transport	Complicated HSP – SPG78; NBIA
*BSCL2*	Seipin	Lipid droplet metabolism/formation	Complicated HSP – SPG17; CMT2; dHMN
*C19orf12*	C19orf12	Unknown	HSP – SPG43; NBIA
*C2orf37*	C2orf37	Unknown	NBIA
*CoASY* ^a^	Coenzyme A synthase	Coenzyme A synthesis	NBIA
*CP*	Ceruloplasmin	Iron oxidation	NBIA
*ERLIN2*	ER lipid raft-protein 2	Cholesterol regulation	Pure/complicated HSP – SPG18
*FTL*	Ferritin light chain	Iron storage	NBIA
*PANK2*	Pantothenate kinase 2	Coenzyme A synthesis	NBIA
*SPART*	Spartin	Trafficking	Complicated HSP – SPG20
*SPAST*	Spastin	Lipid droplet metabolism/formation	Pure/complicated HSP – SPG4
*SPG11*	Spatacsin	Lipid metabolism; lysosomal biogenesis	Complicated HSP – SPG11; CMT; ALS
*SPG15*	Zinc finger FYVE domain-containing protein 26	Lipid metabolism; lysosomal biogenesis	Complicated HSP – SPG15
*WDR45*	WD repeat domain 45	Scaffold protein; autophagy	NBIA
**Mitochondrial-ER contact site**			
*ATL1*	Atlastin1	ER network biogenesis; lipid droplet formation	Pure HSP – SPG3
*MFN2*	Mitofusin 2	ER-mitochondria tether	CMT2A2; HMSN
*REEP1*	Receptor expression enhancing protein 1	ER network biogenesis; lipid droplet formation	Pure HSP – SPG31; dHMN
*SIGMAR1*	Sigma 1 receptor	ER-mitochondria tether	ALS; dHMN
*VAPB*	Vesicle-associated membrane protein B	ER-mitochondria tether	ALS; SMA

^a^Genes that encode enzymes. GPC = glycerophosphocholine; NBIA = neurodegeneration with brain iron accumulation; SPG = spastic paraplegia.

## Wider relevance and treatment perspectives

At the present time, there are no recognized therapeutic interventions for MND or HSP that reverse or prevent motor neuron degeneration. The current pharmacological interventions approved for the treatment of ALS include riluzole and edaravone, which have been shown to slow the rate of disease progression (Brown and Al-Chalabi, 2017). Treatment approaches are largely supportive and aimed at reducing spasticity and improving muscle strength and functional mobility. Importantly, however, there are now a number of interventional studies in the wider group of disorders for which motor neuron degeneration is a key aspect of the disease. A number of these studies have involved targeting the endpoint of lipid imbalance which, in both animal models and humans, and has led to a modulation of disease presentation and/or progression to some extent. Chenodeoxycholic acid (CDCA) treatment is the current standard of care for CTX patients (Salen and Steiner, 2017). CDCA reduces abnormal bile acid synthesis via direct inhibition of the cholesterol 7α‐hydroxylase enzyme and via negative feedback on cholesterol biosynthesis, improving both the biochemical abnormalities and the neurological symptoms if started in the early stages of the disease (Berginer *et al.*, 1984; Salen and Steiner, 2017). Further, there is some evidence to suggest that if CDCA is commenced before the onset of symptoms, neurological symptoms may be delayed or prevented (Berginer *et al.*, 2009). Interestingly, treatment trials of statins in SPG5 patients have consistently revealed a reduction in toxic oxysterol 27-OHC levels; however, unfortunately the correlation and relevance of this finding with clinical outcomes has not been investigated in depth (Mignarri *et al.*, 2014, 2015). Studies of a rat model of CMT1A have revealed a reduction of lipid incorporation into myelin. Substitution of PC and PE in the diet of these rats appears to overcome this myelination deficit, leading to a marked improvement in neuropathic symptoms (Fledrich *et al.*, 2018). Conversely, *Pank2*^−/−^ mice fed a ketogenic diet have been shown to develop a pantothenate kinase-associated neurodegeneration-like syndrome characterized by severe motor dysfunction, neurodegeneration and severely altered mitochondria in the central and peripheral nervous systems similar to that seen in patients with PANK2-associated NBIA. These symptoms can be ameliorated with concomitant pantethine treatment, leading to the suggestion that supplementation with pantethine may be of benefit to patients with pantothenate kinase-associated neurodegeneration (Lamperti *et al.*, 2013).

The development of new methodologies and approaches are important to delineate the specific subcellular biomarker deficits, such as oxysterol and PE imbalance, in HSP and MND. As well as providing potentially powerful biomarkers of disease, such tools may enable monitoring of treatment efficacy of therapeutics to re-address disease-associated lipidome imbalances. Specific genetic subtypes may be more amenable to treatment at targeting (for example) feedback systems, such as CDCA in CTX, or addressing oxysterol imbalance in SPG5. Genetic subtypes of disease leading to more complex subcellular outcomes may require multiple treatment approaches to address the specific mechanistic basis of each condition, and it may be unlikely that one approach will entail a ‘fix all’ treatment. Ultimately, clearer definition of the subcellular lipidome (and other) biological pathways underlying MND and HSP will pave the way for a more elegant approaches for predicting onset and severity of disease, and for designing and monitoring new therapeutic approaches.

## Funding

Grants supporting the studies described include the Medical Research Council (MRC Grant G1002279 to A.H.C.), Newlife Foundation for Disabled Children (to A.H.C. and E.L.B.), and the Wellcome Trust (to A.H.C.), The Hereditary Spastic Paraplegia Support Group UK (to A.H.C. and E.L.B.), and the Halpin Trust (to A.H.C. and E.L.B.).

## Competing interests

The authors report no competing interests. 

## Abbreviations

ALS =: amyotrophic lateral sclerosis
CMT =: Charcot-Marie-Tooth disease
CTX =: cerebrotendinous xanthomotosis
dHMN =: distal hereditary motor neuropathy
HSP =: hereditary spastic paraplegia
MND =: motor neuron disease
PC =: phosphatidylcholine
PE =: phosphatidylethanolamine
PS =: phosphatidylserine
